# Cell-Based Regeneration and Treatment of Liver Diseases

**DOI:** 10.3390/ijms221910276

**Published:** 2021-09-24

**Authors:** Julia Hofmann, Verena Hackl, Hannah Esser, Andras T. Meszaros, Margot Fodor, Dietmar Öfner, Jakob Troppmair, Stefan Schneeberger, Theresa Hautz

**Affiliations:** 1 Department of Visceral, Transplant and Thoracic Surgery (VTT), Center of Operative Medicine, Daniel Swarovski Research Laboratory (DSL) and organLife Laboratory, Medical University of Innsbruck (MUI), 6020 Innsbruck, Austria; julia.hofmann@i-med.ac.at (J.H.); very.hackl@outlook.com (V.H.); andras.meszaros@i-med.ac.at (A.T.M.); margot.fodor@tirol-kliniken.at (M.F.); dietmar.oefner@i-med.ac.at (D.Ö.); jakob.troppmair@i-med.ac.at (J.T.); stefan.schneeberger@i-med.ac.at (S.S.); 2 Centre for Regenerative Medicine, Institute for Regeneration and Repair, The University of Edinburgh, Edinburgh EH16 4UU, UK; h.esser@sms.ed.ac.uk

**Keywords:** liver, cell therapy, stem cells, immunotherapy, regeneration

## Abstract

The liver, in combination with a functional biliary system, is responsible for maintaining a great number of vital body functions. However, acute and chronic liver diseases may lead to irreversible liver damage and, ultimately, liver failure. At the moment, the best curative option for patients suffering from end-stage liver disease is liver transplantation. However, the number of donor livers required by far surpasses the supply, leading to a significant organ shortage. Cellular therapies play an increasing role in the restoration of organ function and can be integrated into organ transplantation protocols. Different types and sources of stem cells are considered for this purpose, but highly specific immune cells are also the focus of attention when developing individualized therapies. In-depth knowledge of the underlying mechanisms governing cell differentiation and engraftment is crucial for clinical implementation. Additionally, novel technologies such as ex vivo machine perfusion and recent developments in tissue engineering may hold promising potential for the implementation of cell-based therapies to restore proper organ function.

## 1. Introduction

The liver is the largest parenchymal organ, which controls a multitude of metabolic functions, including the homeostasis of carbohydrates, lipids, and proteins. It is the primary detoxifying organ [1] and is essential for a proper immune response against pathogens and the further recruitment of innate and adaptive immune cells [1,2]. This can be facilitated by low pressure and flow internal to the sinusoids [3]. Since abundant gut-derived pathogens and exogenous non-pathogenic molecules continuously enter the liver, several mechanisms contribute to prevent unexpected immune responses, establishing a balance between defense and the maintenance of self-tolerance [4,5,6]. The unique histoarchitecture and interactions of the different cell types are responsible for these very specific liver functions.

Despite significant achievements in surgical and pharmacological treatment options, liver diseases, in general, remain a leading cause of death. Severe liver disease resulting in end-stage organ failure may be caused by viral infections, cancer, or genetic predisposition. For many patients, the only treatment option is orthoptic liver transplantation (OLT) [7]. However, OLT is limited by a significant organ shortage, and OLT may not be suitable for specific diseases.

In this review, we first overview the mechanisms underlying the self-regenerating potential of the liver since the development of novel cell-based treatments relies on understanding these processes. Stem-cell-related approaches and cellular immunotherapies are then discussed for the treatment of liver diseases, taking into account ongoing clinical trials and current applications. We further highlight the importance of the chosen route of administration of these therapies and discuss arising novel technologies, which may be advantageous when introducing such innovative treatment protocols.

## 2. Liver Cell Types and Their Potential of Regeneration

### 2.1. Microanatomy of the Liver

**Figure 1:**
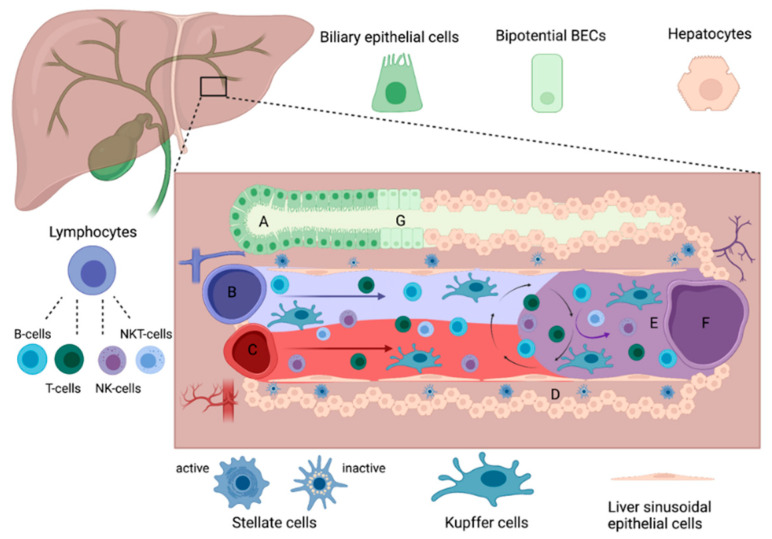
Histoarchitecture of the liver. The portal triad is composed of the bile duct (**A**), the portal vein (**B**), and the hepatic artery (**C**) [8]. The blood flows from the portal vein and hepatic artery through the sinusoids (**E**) towards the central vein (**F**), surrounded by fenestrated LSECs. Lymphocytes and KCs reside intravascular, whereas SCs are found in the space of Disse (**D**) [1]. BECs line the bile duct, where they modify the bile produced by hepatocytes. The junctional region between the bile duct and hepatocytes, known as the canal of Hering (**G**), harbors bipotential BECs [12].

Morphologically, the roughly hexagonal-shaped liver lobes are arranged around the sinusoids, harboring different cells (Figure 1). Every cell type of the liver is characterized by defined responsibilities, which collectively warrant an intact hepatic function at various levels [8]. Parenchymal cells account for 60–80% of the total liver mass. Hepatocytes are the most abundant type within this group, which are organized in cords lining the sinusoids. They play a central role in maintaining organ homeostasis by regulating physiological processes and metabolic activity linked to digestion, including the metabolization of carbohydrates and lipids and the production of bile. The majority of the remaining non-parenchymal cell population (NPC, 20–40% of the total liver mass) is composed of liver sinusoidal endothelial cells (LSECs, 10–20%) that form a permeable barrier between sinusoidal vessels and hepatocytes. Biliary epithelial cells (BECs), which constitute 5% of the total liver mass, will be described in detail in Section 2.1 [9]. Kupffer cells (KCs), also known as intravascular resident macrophages, account for 4–8%. They are located within the sinusoids and primarily perform immunological tasks. Hepatic stellate cells (SCs, 0.2–0.4%) are mesenchymal cells residing in the space of Disse, which is referred to as the space between LSECs and hepatic epithelial cells. SCs remain in a quiescent state; however, when triggered by liver damage, they become activated [1,2]. Moreover, circulating lymphocytes, consisting of T-cells (CD4^+^/CD8^+^), B-cells, natural killer (NK) cells, and NKT cells, are present. They interfere with antigen-presenting cells such as LSECs, KCs, and liver resident dendritic cells (DCs) or directly with hepatocytes, enabling adaptions of the immune response [2,10,11].

### 2.2. Biliary Epithelial Cells—The Lining of the Biliary Tree

The biliary tree is lined by BECs—also known as cholangiocytes [9,13,14,15]. With the enlargement of the bile ducts, the epithelial lining changes: BECs become larger in size, and their architecture changes from cuboidal to columnar. The common bile duct is lined by columnar epithelium with mucus-secreting cells [9,13,14,15].

BECs have several different functions: one of the BECs’ main functions is bile modification and transport. As mentioned above, bile is mainly produced by hepatocytes, although BECs can contribute to the production of the daily bile volume to a limited extent [16,17]. The bile is secreted into bile canaliculi, which are tubules formed between adjacent hepatocytes. They drain into the bile ducts, where the bile is further modified by BECs [1]. This entails a secretion of water and electrolytes (such as HCO_3_^−^ and Cl^−^) as well as the uptake of bile acids, glucose, and amino acids [18,19,20,21,22,23,24]. The secretion of HCO_3_^−^- by BECs and the resulting alkaline environment is known as the “biliary HCO_3_^−^-umbrella”. The HCO_3_^−^ umbrella by the deprotonation of hydrophobic bile salt protects BECs from hydrophobic bile salt toxicity [25,26].

### 2.3. Mechanisms of Liver Cell Regeneration

Since a functioning liver is essential to maintaining body homeostasis, several compensatory regenerative mechanisms exist. Most experimental evidence of liver regeneration is based on animal models of partial hepatectomy. In addition, drug-induced acute liver injury has also been investigated in clinically relevant models of centrilobular liver necrosis and sterile inflammation, such as carbon tetrachloride, thioacetamide, or paracetamol overdose [27]. Both hepatocytes and BECs have been described to proliferate upon acute liver diseases. However, it has been observed that the ability of proliferation in hepatocytes gets lost in chronic diseases, which may also account for NPCs [28]. The molecular mechanism of liver regeneration was extensively reviewed recently [29,30]. Here, we focus on the fundamental mechanisms of liver regenerative potential based on hepatocytes and BECs.

Hepatocytes play a central role in liver regeneration. In this process, several transcription factors trigger mature hepatocytes to re-enter the cell cycle by progressing from the G1 to S phase [31] and, in turn, producing mitogenic growth factors to attract further hepatocytes and other cell types to start proliferating. Such molecules are the vascular endothelial growth factor (VEGF), angiopoietins 1 and 2 (mitogenic for LSECs), transforming growth factor alpha (TGFα) (mitogenic for endothelial cells, LSECs, and SCs), fibroblast growth factor 1 (FGF1) and FGF2 (mitogenic for SCs and LSECs), and the granulocyte–macrophage colony-stimulating factor (GM-CSF) (mitogenic for KCs) [28,32]. Hepatocyte proliferation is mainly controlled by extracellular signals through the tyrosine-protein kinase mesenchymal–epithelial transition factor (MET), which is the hepatocyte growth factor (HGF receptor), and EGFR (epidermal growth factor receptor). Upon hepatectomy, portal venous blood is a major initiator of liver regeneration by the increased shear stress on LSECs and by carrying signaling molecules like EGF, bile acids, and insulin [29,33]. Very early upon partial hepatectomy, the activation of metalloproteinases by the urokinase-type plasminogen activator [34,35] leads to the breakdown of some extracellular matrix components. Most importantly, extrahepatic produced HGF is liberated and activated, soon released in the peripheral blood as well, and provides mitogenic signaling [36]. Quite importantly, HGF or EGFR ligands (EGF, TGFα, heparin-binding EGF-like growth factor, and amphiregulin) induce hepatocyte proliferation in rodents if injected intravenously and in vitro [37]. In addition to HGF, interleukin-6 (IL6), tumor necrosis factor alpha (TNFα), bile acids, noradrenaline, leptin, and serotonin are soon released in the peripheral blood as well, all of which are known to be associated with liver regeneration, termed as auxiliary mitogens. In a uniquely concerted way, only a fraction of hepatocytes will undergo partial dedifferentiation at once, protecting the liver from acute liver failure by sustaining physiologic hepatocyte function in the remaining population [38].

The second key player for the self-regenerating potential of the liver is BECs, which are known as a highly plastic cell population [39]. In rodent models of impaired hepatocyte proliferation or loss of a large amount of liver mass, BECs have been shown to contribute to hepatocyte regeneration by differentiation into hepatocytes [40,41,42]. The plasticity of BECs is facilitated by intermediate transition through bipotential BECs, which have been suggested to be found mainly in the canals of Hering. Bipotential BECs expand during the so-called ductular reaction, which is mainly an expansion of cells of biliary origin observed in chronic liver disease, and can differentiate into both hepatocytes and BECs to enhance liver regeneration [11,43,44,45,46]. Bipotential BECs require NOTCH signaling to differentiate into BECs, while bipotential BEC to hepatocyte differentiation is promoted by Wnt signaling [47,48].

Different molecular pathways such as ten–eleven translocation 1 (TET1, a hydroxymethylcytosine deaminase) mediate changes in the DNA-methylome of BECs, which have been described to be crucial for this cell population to acquire their cellular plasticity [49]. One of the TET1 targets is the hippo-yes-associated protein (YAP)/PDZ-binding motif (TAZ) pathway [49]. YAP has likewise been identified as an important factor for BEC plasticity [39]. YAP mediates BEC self-renewal in liver homeostasis [39,50] and can also be induced by BEC exposure to bile acids [39,50]. This mechanism is hypothesized to contribute to BEC regeneration following acute injury or after partial hepatectomy in rodent models, which is associated with increased bile and bile acid secretion [51,52]. Furthermore, YAP-related upregulation of the biliary marker SRY-Box transcription factor 9 (Sox9) in hepatocytes can initiate hepatocyte-to-BEC differentiation [53,54,55,56].

Taken together, the regenerative potential of acute liver injury or disease is mainly due to hepatocytes re-entering the cell cycle. However, in chronic liver injury, the majority of hepatocytes are senescent [30]; this demands alternative therapy options.

## 3. Cellular Therapies for Treatment of Liver Diseases and Induction of Regeneration

**Figure 2:**
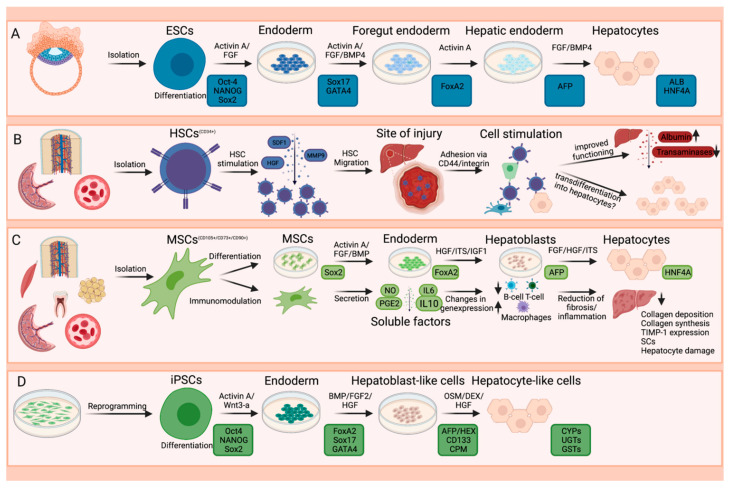
Different sources of stem cells and their mechanism of differentiation for cell-based therapies. ESCs (**A**) are isolated from the early blastocyst [61]. The sources of HSCs (**B**) and MSCs (**C**) include placenta, umbilical cord, bone marrow, and peripheral blood; for MSC, additionally, dental pulp, muscle, and adipose tissue can be utilized [60,83]. iPSCs (**D**) are derived from somatic cells by a reprogramming step. Triggered by different transcription and growth factors, all of them can be differentiated into hepatocytes.

The primary malignancies and underlying circumstances that ultimately lead to liver failure are varied, requiring different therapy strategies. The transplantation of hepatocytes and BECs has been investigated for a variety of diseases, with the most promising results observed for metabolic disorders [57]. To generate those cells in vitro, stem cells are considered the ideal source. They are characterized by their ability of self-renewal and the potential of differentiation into multiple lineages. However, the pluripotency and, thus, the ability to form teratomas in vivo is a major concern and reason why the direct application or transplantation of stem cells has not been clinically approved yet [58]. It can be distinguished by autologous and allogeneic approaches. In the first-mentioned approach, the patient’s own cells are utilized, whereas, in the latter, donor cells are obtained and transferred to the recipient. Thus, allogeneic cell transplantation possesses the potential of immunogenicity. The most frequently used types are embryonic stem cells (ESCs), hematopoietic stem cells (HSCs), induced pluripotent stem cells (iPSCs), and mesenchymal stem cells (MSCs), whereas recent studies have mainly focused on the last two mentioned cells (Figure 2) [59,60]. On the other hand, resident cells of the liver can be directly targeted, whereas immune cells are of special interest within this approach. Especially in cancer, these therapies are utilized for autoimmune diseases and also after transplantation [2,4,5].

### 3.1. Stem Cells: Sources, Mechanism of Action, and Applications

#### 3.1.1. Embryonal Stem Cells (ESCs)

Pluripotent embryonic stem cells possess an unlimited differentiation capacity and can be obtained in the early stages of embryonic development from blastocyst inner cell mass [61,62]. They can be utilized to derive hepatocytes in a four-step protocol following their natural path of development in vivo, which can be monitored through the expression of appropriate gene signatures. Differentiation is driven by interactions with components of several signaling cascades (e.g., activin A, FGF, bone morphogenetic protein 4 (BMP4), HGF) [63,64]. The therapeutic effect of such in-vitro-derived hepatocytes has been investigated in various studies. For example, ESC-derived hepatocytes led to the rescue of acute liver failure induced by acetaminophen in an experimental murine model [61,64]. Moreover, it has been observed that ESC-derived hepatocyte-like cells transport regeneration-promoting trophic factors promoting liver regeneration, and are involved in cell replacement [62]. ESCs may further be used for treatment of fibrosis caused by chronic liver injury. In this mean, the effect of ESC-derived macrophages was tested in a murine model of carbon tetrachloride (CCL4)-induced hepatic injury, which revealed a significant reduction of fibrosis. Simultaneously, fibrogenic myofibroblasts were downregulated, progenitor cells of the liver were activated at the site of injury, and KCs were repopulated. These results give a first insight into the therapeutical usability of ESC-derived macrophages [65]. Besides the application of ESC-derived cell types directly into the liver, the research field that aims to construct liver organoids has gained importance. Liver organoids could serve as an improved model to investigate various liver diseases and develop novel organoid-based therapies [66,67,68,69]. However, its clinical use is associated with ethical concerns due to the deviation procedure of ESCs.

#### 3.1.2. Hematopoietic Stem Cells (HSCs)

Multipotent hematopoietic stem cells are derived from bone marrow, peripheral blood, or umbilical cord blood. In standard isolation protocols, the expression of the surface marker CD34^+^ is used, although it is well known that some of these populations may lack CD34^+^. The migration of the HSCs towards the liver is regulated by the interactions of various chemokines, cytokines, and proteolytic enzymes. In a mouse model, stromal-cell-derived factor 1 (SDF1), matrix metallopeptidase 9 (MMP9), and HGF have been reported to mediate homing to the site of injury [70]. Thereby, adhesion has been reported to be dependent on CD44 and is modulated by C-X-C motif chemokine receptor 3 (CXCR3) and CXCR4 [71]. Moreover, HSCs primed with granulocyte colony-stimulating factor (G-CSF) have been shown to not only selectively migrate to the site of injury but also to induce hepatocyte formation [62,72]. However, the underlying mechanism of differentiation of HSCs is not truly understood [73]. Khurana et al. demonstrated in a cell culture model that one driver is hepatocyte nuclear factor−4α (HNF4α) [74]. In further in-vitro studies, HGF [75,76], FGF2, and FGF4 [75] have also been identified as key players. On the other hand, several studies support the theory of cell fusion rather than differentiation [77,78,79]. Nevertheless, in animal but also clinical studies, reasonable results could be achieved. In a fibrotic mouse model induced by CCL4, the expression of MMP9 and MMP14, which are involved in the degradation of collagen fibers, resulting in a reduction of fibrosis, was observed. Moreover, MMP9 secrets HSC differentiation and proliferation stimulating factors (e.g., c-Kit) to reduce inflammatory reactions and also triggers movement towards the afflicted tissue [80]. In a clinical trial, 48 patients suffering from end-stage liver disease, either from hepatitis C (HCV) infection or due to autoimmunity, received injections of autologous CD34^+^ HSCs. Liver function significantly improved according to serum levels of albumin, the international normalized ratio (INR), and transaminases during 12 months of follow-up [81]. In a larger study including 90 patients, the effect was even enhanced by repeated CD34^+^ HSCs injections. A linear enhancement of the effect was reported during the 12-month follow-up, suggesting engraftment of the cells; however, the study lacked histological proof [82].

#### 3.1.3. Induced Pluripotent Stem Cells (iPSCs)

A special feature of iPSCs is their production by reprogramming adult somatic cells back to a pluripotent state. iPSCs may then be utilized to produce various cell types, including hepatocytes and BECs. To generate functional hepatocytes, phase-specific markers, growth factors, cytokines, and chemicals are necessary within a three-step transformation from human iPSCs to endodermal cells, hepatoblast-like cells (HLC), and, finally, differentiated hepatocyte-like cells. The majority of protocols include the activation of Activin A for endoderm differentiation. As a next step, treatment with BMP and FGF2 induces differentiation into HLCs, and, finally, HGF and oncostatin M (OSM) trigger maturation. Such hepatocyte-like cells are defined by the expression of hepatocyte-specific markers and, for example, are used to treat end-stage liver disease by repopulating damaged liver tissue [62,84]. In a murine model for cell replacement therapy, the therapeutic potential of transplanted iPSC-derived HLCs was examined in carbon-tetrachloride-induced liver injuries. The created hepatic lineage expressed hepatocyte-specific markers, stored glycogen and lipids, and stimulated metabolic activity. One week after transplantation, the incorporation and functional integration of HLCs into the liver was evident, leading to enhanced albumin serum levels and decreased levels of lactate dehydrogenase and bilirubin. Further to this, these mice showed improved survival and slightly reduced fibrosis during a period of 5 weeks after transplantation compared to untreated controls [84]. In another mouse study, hepatocyte-like cells integrated with an albumin reporter (referred to as iHep) produced from iPSCs were transplanted into fibrotic livers, which resulted in beneficial effects including a decrease in thioacetamide-induced fibrosis, liver cell apoptosis, and pro-inflammatory factors and improved liver function. Moreover, the number of proliferating hepatocytes and iHeps expressing albumin was elevated following transplantation, demonstrating their potential to promote regenerative processes [85]. Besides the creation of HLCs, human iPSCs are also used to generate mature KCs. Therefore, iPSCs are differentiated into macrophage precursors, which are then used to generate the induced KCs. These cells mostly displayed identical gene expression patterns and functional properties compared to primary adult human KCs. At the current stage of research, induced KCs are used in different co-culturing models (e.g., with hepatocytes, macrophage precursors, or iHeps) in vitro to study inflammation, test for hepatoxicity, or examine cholestasis formation [86]. In cell-free therapies, iPSCs are used for the production of EVs. In a murine model of liver injury, the injection of EVs displayed anti-fibrotic effects by reducing profibrogenic factors or inhibiting the profibrogenic activity of human stellate cells. These results indicate that iPSC-derived EVs may exert therapeutic potential for the treatment of chronic liver diseases where the formation of fibrosis is the main feature [87].

#### 3.1.4. Mesenchymal Stem Cells (MSCs)

MSCs are multipotent stem cells and must fulfill at least the following criteria stated by the Society for Cellular Therapy [88]. First, MSCs must be plastic-adherent when maintained in standard culture conditions. Second, MSCs must express CD105, CD73, and CD90 and lack expression of CD45, CD34, CD14 or CD11b, CD79α or CD19 and HLA-DR surface molecules. Third, MSCs must differentiate into osteoblasts, adipocytes, and chondroblasts in vitro. They are further known for their high differentiation potential and low immunogenicity and can be isolated of different tissues, including bone marrow (bm), adipose tissue, muscle, peripheral blood, placenta, umbilical cord (uc), and dental pulp (dp) [83]. Differentiation into both hepatocytes and BECs has been described using different protocols in vitro with the use of specific factors such as HGF, FGF4, insulin-transferrin-sodium selenite (ITS), and insulin growth factor 1 (IGF1) [89,90,91]. However, the differentiation was not limited to in vitro studies. In a mouse model of biliary injury, the administration of bmMSCs led to the restoration of the injury through delta ligand-like 4 (DLL4) activation, which enhanced BEC differentiation [92]. Iwanaka et al. injected dpMSCs into CCL4-induced chronic liver fibrotic mice, which induced tissue regeneration due to in vivo differentiation [93].

Besides the differentiation into hepatocytes and BECs for tissue replacement, the regenerative effect of MSCs may mainly underlie paracrine effects. Upon administration, MSCs migrate to the injured tissue area and secrete growth factors related to liver regeneration, which, in turn, stimulate cell proliferation, enhance angiogenesis, and restrain apoptosis [62,94,95]. Furthermore, MSCs promote liver regeneration by immunomodulation due to the release of soluble factors (e.g., nitric oxide (NO), prostaglandin E2 (PGE2), IL6, or IL10). This, in turn, leads to the downregulation of T-cells, the inhibition of B-cells, or the advantageous modulation of liver injury, hence improving inflammatory macrophages. Additionally, MSCs possess immunosuppressive properties regulated by cytokines (e.g., IL1α, TNFα, or interferon gamma (IFNγ)) and interactions with chemokines (e.g., XCR4/5/7) [96,97,98]. Moreover, upon the transplantation of MSCs, anti-fibrotic effects have been reported. Quiescent SCs can differentiate into fibrogenic myofibroblasts in chronic liver injury, but due to the secretion of several trophic factors such as TNFα and IL10, collagen synthesis is attenuated. On the other hand, HGF and NGF induce the apoptosis of SCs [97,99]. Ultimately, anti-inflammatory reactions are also promoted by MSCs, which may reduce hepatocyte damage [97,98]. In a clinical trial of 43 patients suffering from acute-on-chronic liver failure caused by hepatitis B (HBV) infection, all 24 patients in the test group treated with ucMSCs showed an enhanced survival rate, increased albumin secretion, and reduced transaminases levels compared to the control group [100]. Analogously, favorable results have also been reported for ucMSCs in a clinical study on primary biliary cirrhosis [101]. Recently, MSCs are a prominent tool to create EVs such as apoptotic bodies or exosomes in cell-free therapies. Specifically, cell-type-specific exosomes containing bioactive molecules such as various RNA types, other nucleic acids, proteins, and lipids are widely used in therapeutical trials due to their advantageous functions (e.g., involvement in drug delivery, immune regulation, tissue repair/regeneration, angiogenesis, cell proliferation, collagen reduction, or metabolic activities). Current studies mainly focus on the therapeutic effects of bm- or uc-derived MSC-EVs on acute liver failure, liver fibrosis, and hepatocellular carcinoma treatment using cell culture or experimental models. Thus, further studies are needed to assess their applicability in humans [98,102].

Taken together, stem cells as a source for hepatocytes and various progenitors, which can be utilized for subsequent transplantation, show great potential. Numerous clinical trials on hepatocyte transplantation have been conducted or are ongoing currently; however, no treatment has been clinically approved to date. The majority of these studies have reported promising short time efficacies, but they could not maintain the efficacy long term [57]. The indications for hepatocyte transplantation include acute and acute-on-chronic liver failure [103]. Still, the majority of reports are on inherited metabolic disorders, including different types of urea cycle defects [104,105,106,107,108] and Crigler-Najjar syndrome [109,110,111]. However, randomized trials are lacking; thus, it is difficult to conclude on the efficacy [112]. On the other hand, stem cells may also be administered in an undifferentiated manner to exert their effect. For this purpose, MSCs are considered the most promising candidate since numerous clinical trials are currently ongoing. Recently, Smets et al. reported a safe and effective treatment of MSC therapy in 14 pediatric patients suffering from metabolic disorders [113]. They utilized a specifically manufactured cell suspension called HepaStem, which is also currently investigated for patients with acute-on-chronic liver failure (NCT04229901). In general, MSC-based therapies are considered safe, but there are also reports of side effects [102,114], indicating the need for further studies. Another challenge in the context of stem cell therapies, in general, is to enable cell engraftment, which has been reported to be rather poor in some studies [57,103].

### 3.2. Immune Cell Therapies: A Novel Paradigm in the Development of Therapies

#### 3.2.1. Regulatory T-Cells (Tregs)

Regulatory T-cells (Tregs) play multiple roles in the immunopathology of liver diseases. First of all, they are crucial in maintaining hepatic immunological self-tolerance. Normal liver contains a low frequency of CD8^+^ or CD4^+^ effector T-cells and Tregs; however, inflammatory and autoimmune liver diseases are associated with enrichment of these subsets of lymphocytes [115,116]. Tregs attenuate inflammation by suppressing effector T-cell proliferation and cytokine secretion, thereby avoiding chronic lobular or interface hepatitis, which may result in cirrhosis, liver failure, or hepatocellular carcinoma (HCC) [117]. Accordingly, deficiencies in Treg cell development or function result in uncontrolled immune responses and tissue destruction, leading to inflammatory disorders such as graft-versus-host disease (GvHD), transplant rejection, and autoimmune diseases. Tregs have been considered an attractive therapeutic candidate for restoring immune tolerance in autoimmune and autoinflammatory diseases and in the course of transplantation of hematopoietic stem cells and solid organs, with the intent to reduce or replace immunosuppressive drugs [118]. Overall, Treg cell infusions have been shown to be safe, even if the manufacturing of these cells in the context of chronic immunosuppressive treatments remains challenging. Thus, enhancing Treg frequency, along with administration of alloantigen-specific Tregs, may potentially be a future therapeutic option to maintain post-transplant tolerance [116,119].

Moreover, evidence suggests that a quantitative or functional deficiency in Tregs plays a key role in the pathogenesis of autoimmune hepatitis (AIH) and other autoimmune diseases [116,120,121]. In AIH, the pathogenetic effector CD4^+^ and CD8^+^ T-cell immune response to auto-antigen causes a loss of immunological tolerance to hepatocytes [116]. The standard treatment to achieve remission is represented by long-term immunosuppression; however, progression to end-stage liver disease occurs in 10–20% of patients [116]. Consequently, a shift towards regulatory dominance by applying cellular therapy to enrich hepatic Tregs could be a therapeutic strategy for restoring self-tolerance [116]. Targeting CXCR3, which is highly expressed in peripheral Tregs and enriched in inflamed liver tissue, as well as selectively expanding Tregs by IL2 or IL2/anti- IL2 complex administration, may be potential options to suppress ongoing hepatitis [116,122,123]. Additionally, Tregs were administered to protect against fulminant hepatitis by suppressing effector T-cells in mouse acute HBV infection [124]. The field is steadily moving forward, with several phase I clinical trials aiming to evaluate Treg cell therapy in AIH (NCT02704338) and liver transplantation [116,125]. Immunotherapy was investigated as a further therapeutic option for patients with HCC. On the other hand, FOXP3^+^ Tregs are one of the mechanisms of tumor-driven immune evasion due to their capacity to block tumor-specific CD8^+^ and CD4^+^ effector T-cells. Hence, attenuating Tregs may promote anti-tumor immunity [126]. The administration of blocking antibodies such as daclizumab, which reduces FOXP3^+^ CD25^high^ CD45RA^neg^ Treg numbers, or cytotoxic T-lymphocyte-associated antigen 4 (CTLA4), thereby depleting CD25^+^ Tregs, represents potential approaches to do so [127,128]. Tempering successful immunotherapy by degrading local Tregs while preserving host immune response may improve HCC therapy.

#### 3.2.2. Chimeric Antigen Receptor-Engineered T-Cells (CAR-Ts)

Chimeric antigen receptor-engineered T-cell (CAR-T) therapy is a promising strategy in the development of novel and personalized cancer therapies; it is based on T-cell transfer after the stimulation, expansion, and reinfusion of peripheric antigen-specific T-cells derived from patient blood. So far, CAR-T therapy has been investigated in clinical trials to treat hematological diseases and solid tumors [4]. These cells, which are engineered to recognize specific tumor-associated antigens (TAA), consist of four components: (i) the external component, usually made by the single-chain variable fragment (scFv) domain; (ii) the space region or hinge, consisting of the IgG1 hinge-CH2-CH3 Fc domain connecting the scFv; (iii) the transmembrane region; and (iv) the intracellular signaling domain, which varies from one CAR-T generation to another [129]. Various CAR-T approaches for the treatment of HCC or HCC metastases or against different TAAs have been evaluated in pre-clinical models, showing increased anti-tumor efficacy. However, a lack of specific tumor antigens, limited trafficking, and the penetration of CAR-T cells to tumor sites, as well as the immunosuppressive tumor microenvironment, are still major hurdles to overcome [4]. Moreover, CAR-T therapy is associated with substantial side effects, such as tumor lysis syndrome and cytokine release syndrome. Both complications may be managed by enhancing the selectivity of CARs and controlling CAR-T activity in the future [130]. Currently, 22 clinical trials are investigating the application of CAR-T for HCC treatment, most of them targeting glypican 3 (GPC3), Mucin 1 (MUC), and carcinoembryonic antigen. To date, no clinical trials investigating the efficacy of CAR-T therapy to treat HCC have been completed [129,131,132].

#### 3.2.3. Dendritic Cells (DCs)

DCs are bone-marrow-derived antigen-presenting cells, promoting self-tolerance in the healthy steady-state while regulating innate and adaptive immunity [133]. Recent approaches have demonstrated a role for infused regulatory DCs (DCreg) in reducing T-cell activation and coordinating CD4^+^ and CD8^+^ memory T-cell responses [134], particularly for the therapy of allograft rejection. The first in-human, single-center, open-label phase I/II study has been initiated to test the safety of a single infusion of donor-derived DCreg in de novo adult living donor liver transplant recipients. Furthermore, the impact of DCreg as a novel adjunct induction therapy to induce donor-specific T-cell hyper-responsiveness and to allow early withdrawal of immunosuppressive therapy will be tested in this study [135]. Strategies selectively targeting DCs to enhance their immune regulatory function still promise a potential therapeutic application [133].

#### 3.2.4. Macrophages, Monocytes, and Kupffer Cells (KCs)

Hepatic macrophages are key players in various types of liver diseases and are delineated as promising targets to develop new therapies [136]. These include resident KCs and monocyte-derived macrophages (MoMFs). While KCs represent the main hepatic macrophages involved in homeostasis, hepatic damage results in massive infiltration of MoMFs into the injured liver, which contributes to augmenting liver inflammation, fibrosis, and HCC [137]. The targeting of hepatic macrophages by chemokines in order to regulate their recruitment, differentiation/polarization, and activation has been investigated in pre-clinical models [138]. Additionally, therapies based on inhibiting the intracellular inflammatory signaling pathways of KC have been explored [139]. The expression of Cadherin 11 (CDH11) in injured cells has been shown to regulate myofibroblast activation during the development of fibrosis. Consequently, CDH11 may represent the main target of macrophages for the treatment of liver fibrosis [140]. Moreover, approaches to limit monocyte recruitment to the liver, mostly based on interfering with the chemokine signaling for monocytes, have been investigated. Given the categorization of MoMFs in Ly6C^high^ monocytes, causing organ impairment and Ly6C^low^ monocytes, which are restorative, a suggested strategy was to switch Ly6C^high^ monocytes into Ly6C^low^ monocytes in order to achieve the restoration of proper organ function [141]. Taken together, the significant role of macrophages in liver diseases highlights them as ideal targets when developing novel cell-based therapies; however, the heterogeneity of this cell population represents a challenge in this context.

In summary, the most promising efforts in developing immune cell therapies to treat and cure liver diseases have been reported for the treatment of HCC. Several clinical phase I/II studies are currently ongoing, with most of them investigating the potential of CAR-T cell therapies [142].

## 4. Technological Developments for Improved Delivery and Engraftment of Cell-Based Therapeutics

Several factors have to be considered when developing cell-based therapies. A major hurdle is the route of administration since this is a critical parameter for the efficacy of such therapies. For in vivo injection, it can be distinguished between local and systemic delivery. The most prominent route is by portal vein or hepatic artery infusion; however, ectopic implantation, either into the spleen or peritoneum, has also been reported. It is important that the cells reach their target—the liver parenchyma—within 24 h since, otherwise, they will be cleared by macrophages [57,60,143]. Thus, the use of matrix components such as hyaluronic acid (HA), collagen, and laminin has been studied by different groups to enhance engraftment [144]. Nevi et al. coated hBTSCs with HA and administered those via the spleen into immunocompromised mice. Compared to the control group, the efficacy of the engraftment could be increased four-fold. Moreover, metabolic function according to serum albumin levels was also improved [145].

Recent advances in organoid technologies as a state-of-the-art 3D culture model are not only of interest to study diseases or for pharmacological screening but may also be utilized to be transplanted directly to improve organ function, as shown by a few experimental studies. For example, Kuijk et al. generated liver organoids of rat liver stem cells and subsequently transplanted them into a rat model of acute liver failure. In 7 out of 11 rats, proper cell engraftment was reported with improved liver function, as indicated by albumin secretion [146]. In another study, extrahepatic cholangiocyte organoids were able to reconstruct the biliary tree in a mouse model of extrahepatic biliary injury [147].

Nevertheless, OLT will most likely remain the only treatment/curative option for a variety of end-stage liver diseases in the near future. Thus, ex vivo treatment of marginal organs may help to overcome organ shortage. Machine perfusion of organs is not only a versatile tool for organ preservation but may also be utilized for the delivery of cells to promote liver regeneration and repair. In this context, normothermic machine perfusion (NMP) is of special interest. The main blood vessels of the liver are thereby cannulated to maintain a perfusion circuit, which is carried out at 37 °C to achieve close to physiological conditions. The therapeutic agents can be added directly into the perfusate to be delivered into the vasculature of the organ. The group of Yang et al. administered bmMSCs in a donation after circulatory death (DCD) rat model during NMP, which resulted in improved liver function, reduced histological damage, and restored mitochondrial function [148]. In another murine study, the supplementation of the perfusate with EV derived from human liver stem cells led to improved liver function compared to the control group [149]. Laing et al. perfused discarded human organs under normothermic conditions and delivered multipotent adult progenitor cells to the organs. They not only showed engraftment of the cells via confocal microscopy but, additionally, three out of six organs met the viability criteria for transplantation [150]. However, only a limited number of experiments have been reported for the liver so far, in contrast to other organs such as kidney and heart.

Recently, Sampaziotis et al. combined two of the most promising technologies to repair human livers declined for transplantation due to ischemic bile duct injury, indicated by a pH < 7.5: red fluorescence protein (RFP)-expressing cholangiocyte organoids (mechanically dissociated, 10 × 10^6^ cells) were injected into the terminal branch of the intrahepatic ducts of three human livers at the start of NMP; at the same time, they were preserved at the perfusion device ex vivo for up to 100 h. In this experimental effort, the successful engraftment of REP organoids into the intrahepatic biliary tree was reported for the very first time when combining machine perfusion and 3D cell culture techniques. The organoids expressed key biliary markers and did not differentiate into other lineages. After 100 h of NMP, no signs of cholangiopathy were found [151]. While these promising results represent the very first step towards revolutionizing the concept of treating and even curing severe liver diseases, it may take another decade to fully implement this therapeutic option in clinical routine, leaving many questions unanswered at this point.

## 5. Conclusions

Cellular therapies are a promising alternative that may replace orthoptic liver transplantation for a variety of liver diseases in the future or at least bridge the time to transplantation. Interdisciplinary efforts are made to enhance their efficacy by combining recent achievements in the fields of tissue engineering and machine perfusion, which may enhance the number of organs for transplantation and may also enable the regeneration of the patients’ own organs in the future. The concept of ex vivo organ treatment and regeneration is not limited to liver diseases but can also be applied to other organs such as heart, kidney, and lung. A better understanding of the underlying mechanisms of the various diseases, together with drug engineering approaches, has led to the development of novel immunomodulatory drugs, which is a great achievement towards personalized medicine.

## Abbreviations

AFP	Alpha-1-fetoprotein
AIH	Autoimmune hepatitis
BEC	Biliary epithelial cells
bm	Bone marrow
BMP	Bone morphogenic protein
CAR-T	Chimeric antigen receptor-engineered T cells
CCL4	Carbon tetrachloride
CDH11	Cadherin 11
CPM	Carboxypeptidase M
CTLA4	Cytotoxic T lymphocyte-associated antigen 4
CXCR	C-X-C motif chemokine receptor
DC	Dendritic cells
DCreg	Regulatory DC
DEX	Dexamethasone
DLL4	Delta-ligand like 4
dp	Dental pulp
EGFR	Epidermal growth factor receptor
ESC	Embryonic stem cell
EV	Extracellular vesicles
FGF	Fibroblast growth factor
FoxA2	Forkhead-box protein A2
G-CSF	Granulocyte colony-stimulating factor
GATA4	Gata-binding protein 4
GM-CSF	Granulocyte-monocyte colony-stimulating factor
GPC3	Glypican 3
GvHD	Graft-versus-host disease
HA	Hyaluronic acid
HBC	Hepatitis B
HCC	Hepatocellular carcinoma
HCV	Hepatitis C
HGF	Hepatocyte growth factor
HLC	Hepatoblast-like cells
HNF4α	Hepatocyte nuclear factor 4α
HSC	Hematopoietic stem cell
IGF1	Insulin growth factor 1
iHep	Induced hepatocyte-like cells
IL	Interleukin
INFγ	Interferon gamma
INR	International normalized ratio
iPSC	Induced pluripotent stem cell
ITS	Insulin-transferrin-sodium selenite
KC	Kupffer cells
LSEC	Liver sinusoidal endothelial cells
MMP	Matrix metallopeptidase
MoMF	Monocyte-derived macrophage
MSC	Mesenchymal stem cell
MUC	Mucin 1
NMP	Normothermic machine perfusion
NO	Nitric oxide
NPC	Non-parenchymal cells
OLT	Orthoptic liver transplantation
Oct4	Octamer binding transcription factor 4
PEG2	Prostaglansin E2
RFP	Red flourescene protein
SC	Hepatic stellate cells
scFv	Single chain variable fragment
SDF1	Stromal-cell-derived factor 1
Sox	SRY-Box Transcription Factor
TAA	Tumor-assocated antigens
TAZ	PDZ-minding motif
TET1	Ten-eleven translocation 1
TGFα	Transforming growth factor alpha
TIMP1	Inhibitor of metalloprotease 1
TNFα	Tumor necrosis factor alpha
Tregs	Regulatory T cells
uc	umbilical cord
VEGF	Vascular endothelial growth factor
YAP	Yes-associated protein
